# Acid-Free Synthesis of MIL-101/GO Composites with Ultrahigh Selectivity for Adsorptive Separation of C_3_F_8_ from N_2_

**DOI:** 10.3390/ma19040753

**Published:** 2026-02-14

**Authors:** Ziyang Yang, Xicheng Sun, Wenhui Yuan, Li Li

**Affiliations:** 1 School of Chemistry and Chemical Engineering, South China University of Technology, Guangzhou 510640, China; 2 Guangdong Engineering Technology Research Center of Advanced Insulating Coating, South China University of Technology-Zhuhai Institute of Modern Industrial Innovation, Zhuhai 519175, China; 3 Guangdong Guangda New Energy Technology Co., Ltd., Guangzhou 573100, China; 4 School of Environment and Energy, South China University of Technology, Guangzhou 510006, China

**Keywords:** MIL-101(Cr), GO, C_3_F_8_, adsorption

## Abstract

**Highlights:**

**What are the main findings?**

- A green acid-free route produces MIL-101/GO-0.1, enabling superior C_3_F_8_ capture.
- The content of graphene oxide affects the adsorption properties of the composite material.
- MIL-101/GO-0.1 achieved a record-breaking IAST separation selectivity of 17,069 for C_3_F_8_/N_2_.

**What are the implications of the main findings?**

- Overcome the current technical bottleneck of low C_3_F_8_ adsorption capacity.
- Green synthesis coupled with high adsorption capacity opens up possibilities for industrial applications.
- This provides insights for future research into the adsorption and separation of C_3_F_8_.

**Abstract:**

As a perfluorinated compound with a high global warming potential, octafluoropropane (C_3_F_8_) needs to be efficiently separated from industrial waste gas, but separating it from nitrogen at low concentrations is highly challenging. To address the common drawback of using corrosive acids in conventional MIL-101(Cr) synthesis, this study developed a green, acid-free solvothermal method for preparing graphene oxide (GO)-modified MIL-101(Cr) composites (MIL-101/GO). By systematically varying the GO doping, the optimal composite (MIL-101/GO-0.1) exhibited breakthrough adsorption performance: its equilibrium adsorption capacity for C_3_F_8_ reached 210 mg/g in fixed-bed breakthrough experiments. The predicted C_3_F_8_ adsorption selectivity relative to N_2_ reached 17,069, ranking among the highest values reported for adsorbents, indicating a significant performance enhancement over the pristine MIL-101(Cr). Mechanistic analysis reveals that graphene oxide not only increases specific surface area and micropore volume but also enhances dispersion forces, substantially boosting affinity for C_3_F_8_. Additionally, the composite exhibits outstanding cycling stability and thermal stability. This study provides a novel eco-friendly synthetic strategy for high-performance metal–organic frameworks and offers a highly promising candidate material for industrial-scale fluorocarbon recovery.

## 1. Introduction

C_3_F_8_, also known as perfluoropropane, results from sulfur hexafluoride breakdown, an arc-suppression compound, when it comes into contact with equipment exposure high-voltage electric fields. The existence of this compound does not just degrade the longevity of electrical insulating gases—it also significantly worsens climate change when emitted. With a staggering global warming potential reaching 8900, C_3_F_8_ is approximately 8830 times more potent than carbon dioxide in trapping atmospheric heat [1,2]. Furthermore, existing reports on C_3_F_8_ removal are scarce. Therefore, effective capture and recovery are important for reducing greenhouse gas emissions, protecting the environment, and realizing resource recycling.

Among the existing methods for C_3_F_8_ treatment, adsorption technology is widely used due to its simplicity and safety, and efficient adsorbents are the core of adsorption technology [3,4,5]. However, reports on adsorptive separation of C_3_F_8_ from nitrogen remain extremely limited. Therefore, exploring novel adsorbents with enhanced C_3_F_8_ uptake represents a valuable research direction.

Metal–organic framework (MOF) composites have been extensively investigated due to their boosted adsorption capabilities when mixed with other substances like carbon nanotubes [6], graphene oxide [7], metals [8], and activated carbon (AC) [9]. Among these, graphene oxide (GO) is recognized as a highly effective component for improving gas adsorption, mainly because its tightly packed atomic structure and wealth of oxygen-containing functional groups not only enhance dispersive interactions but also effectively enhance material porosity through doping into the composite. Bandosz and crew [10,11,12,13] prepared MOF (MOF-5, HKUST-1, and MIL-100 (Fe))/GO composites and put them through their paces with small molecular gases (NH_3_, H_2_S, NO_2_). They discovered that composites exhibit notably enhanced adsorption capabilities for these gas molecules. Chen et al. [7] prepared MOF-505/GO, and the adsorption capacity of the composites for CO_2_ was greatly increased as compared to the parent MOF-505, and the water stability of the composites was also superior to that of the parent MOF-505. MIL-101 remains among the most porous known materials [14]. And it serves as a key representative of the investigated MOFs. With its exceptional adsorption capabilities, MIL-101 stands out as a promising material for gas molecule capture. Building on this potential, Sun et al. [15] developed MIL-101(Cr)/graphene oxide that outperformed the original framework. These composite materials possessed significantly enhanced surface area and pore volume, leading to superior n-hexane adsorption performance. The study found that incorporating 5 wt% GO into MIL-101(Cr) yielded optimal results, producing composites with both the maximum specific surface area and the highest n-hexane uptake capacity.

However, hydrofluoric acid with strong corrosiveness was used in the traditional MIL-101 preparation, and there are fewer reports for this material to be applied in the adsorption and separation of C_3_F_8_ and nitrogen mixtures. Based on the above considerations, the objective of this study is to prepare a composite material of MIL-101 and graphene oxide (MIL-101/GO) in an acid-free environment and investigate its adsorption and separation performance for C_3_F_8_ and nitrogen mixtures. This study will examine how MIL-101 and composites interact with trace amounts of C_3_F_8_, focusing specifically on adsorption characteristics. Additionally, the research will explore how incorporating graphene oxide influences the composite’s structural properties, crystalline arrangement, thermal resilience, and overall adsorption efficiency.

## 2. Materials and Methods

### 2.1. Materials

Chromium (III) nitrate hydrate (Cr (NO)_3_·9H_2_O, AR 99%), graphene oxide (GO), and terephthalic acid (H_2_BDC, 99%) were all purchased from Shanghai Aladdin Biochemical Technology (Shanghai, China), and the solvents of N,N-dimethylformamide and ethanol were obtained from Guangdong Guanghua Science & Technology Co., Ltd., Shantou, China; C3F8 was purchased from Wuhan Newlad Specialty Gases Co., Ltd., Wuhan, China.

### 2.2. Synthesis of MIL-101(Cr)

**Figure 1:**
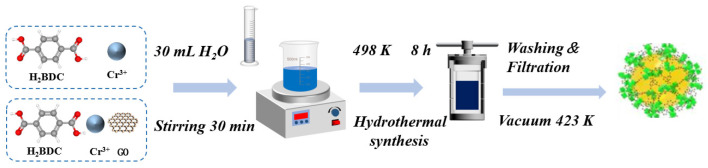
Synthesis process diagram of the obtained sample.

Figure 1 shows the synthesis process diagram of the sample. The MIL-101(Cr) was synthesized in an acid-free environment. Firstly, 2.4 g of chromium nitrate hydrate was dissolved in 20 mL of deionized water, while 1 g of H_2_BDC was separately dissolved in 10 mL of DMF. Both mixtures were stirred vigorously for 30 min to ensure complete dissolution before being combined and transferred to a reactor and reacted at 493 K for 8 h. This temperature was selected to provide sufficient activation energy for framework assembly while avoiding decomposition, and the duration was optimized to ensure complete crystallization, as confirmed by preliminary time-dependent experiments. Centrifuge the resulting mixture to obtain a green solid product. In order to obtain a purer, highly porous, and extensive-surfaced sample, the product underwent three washes using N,N-dimethylformamide and methanol. Finally, drying at 423 K for 12 h to obtain the MIL-101 sample.

### 2.3. Synthesis of MIL-101/GO

The MIL-101/GO composites were synthesized following a procedure analogous to the standard MIL-101preparation. Graphene oxide was incorporated into the precursor solution at varying concentrations—0.1 wt%, 0.2 wt%, 0.5 wt%, and 1 wt% by weight relative to the starting material. This mixture was then subjected to ultrasonication in a water bath at 298 K for 30 min to fully disperse the GO sheets, minimizing agglomeration. These final products are labeled as MIL-101/GO-n, with “n” corresponding to the respective GO loading percentages (0.1, 0.2, 0.5, and 1).

### 2.4. Characterization

The morphology of the synthesized materials was examined using a TESCAN MIRA LMS (Brno, Czech Republic) scanning electron microscope (SEM). Prior to imaging, the powder samples were sputter-coated with a thin layer of gold to enhance conductivity. Crystal structure and phase purity were analyzed by X-ray diffraction (XRD) on a Rigaku Smart Lab SE diffractometer (Rigaku, Tokyo, Japan). Patterns were recorded in the 2θ range of 10° to 40° with a scanning speed of 5°/min. The functional groups present in the materials were identified using a Thermo Fisher Scientific Nicolet iN10 Fourier transform infrared (FT-IR) spectrometer (Thermo Fisher Scientific, Waltham, MA, USA) in transmission mode. Spectra were collected over the wavenumber range of 2400–400 cm^−1^ with a resolution of 4 cm^−1^ and averaged over 32 scans. The thermal decomposition behavior of the samples was examined using the TA Q500 thermogravimetric analyzer (TA Instruments, New Castle, DE, USA), with temperature ramping from 303 K to 873 K at 10 K per minute under nitrogen flow. To investigate the porous structure, including specific surface areas, pore sizes and volume, nitrogen physisorption measurements were conducted at 77 K on a Micromeritics ASAP 2460 system, which comes with integrated data processing and analysis capabilities for comprehensive surface characterization.

### 2.5. C_3_F_8_ Adsorption Experiments

Adsorption isotherms for C_3_F_8_ were obtained for MIL-101/GO-n on a BSD-660C A6C weight analyzer (BSD Instrument, Beijing, China). The weight of the sample for each run was approximately 200 mg. Beforehand, the sample underwent thorough degassing within the BSD-660C A6C chamber, first being evacuated and subsequently held at 403 K for 10 h to ensure complete removal of any adsorbed contaminants.

The breakthrough curve of C_3_F_8_ was tested by laboratory self-assembled equipment. To simulate low-concentration C_3_F_8_ adsorption, the nitrogen-to-C_3_F_8_ mixture was maintained at a 400:1 ratio.

## 3. Results

### 3.1. Structural Analysis and Characterization

**Figure 2:**
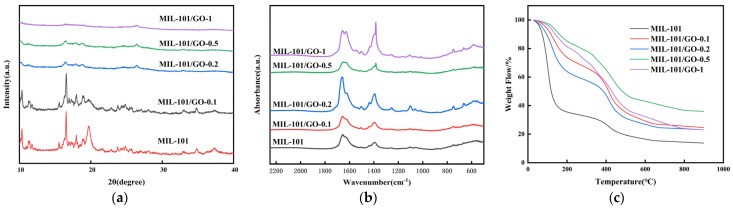
(**a**) X-ray diffraction patterns of MIL-101 and MIL-101/GO-n, (**b**) Fourier transform infrared spectra of MIL-101 and MIL-101/GO-n, (**c**) thermogravimetric analysis (TGA) of MIL-101 and MIL-101/GO-n.

Figure 2a shows the powder X-ray diffraction patterns of the MIL-101 and MIL-101/GO-n samples. The near-identical diffraction profile of MIL-101/GO-0.1 to its parent material demonstrates that MIL-101 remains the dominant phase while maintaining its crystalline integrity in the composite form. However, as the graphene oxide content increases across the series (MIL-101/GO-0.2, MIL-101/GO-0.5, and MIL-101/GO-1), we observe a marked attenuation in peak intensity. This progressive weakening of diffraction signals likely stems from substantial structural deformation of the MIL-101 framework caused by excessive incorporation of GO, as supported by reference [16].

Figure 2b shows the Fourier transform infrared spectra (FTIR) of the MIL-101 and composites. The spectra exhibit close similarity across MIL-101 and its composites. Typically, the frequency of the carboxylate stretching vibration and its difference (Δ = νas (COO-) − νs (COO-), where νas (COO-) denotes the asymmetric stretching vibration frequency and νs (COO-) denotes the symmetric stretching vibration frequency) serve as spectroscopic indicators for identifying carboxylate bonding configurations [17]. The original asymmetric stretching vibration peak (COO-) and symmetric stretching vibration peak (COO-) of H_2_BDC appear at 1681 cm^−1^ and 1285 cm^−1^ [18], respectively. Due to changes in the vibration frequency of the carboxyl group, these peaks shift to 1544 cm^−1^ and 1396 cm^−1^. This phenomenon may be due to the coordination interaction between the carboxylate and the Cr^3+^. The calculated Δ value is 148 cm^−1^. Generally, the carboxylate-metal cation binding mode is identifiable through Δ values [18]. When Δ > 200 cm^−1^, it is expected to be a monodentate ligand; when Δ < 110 cm^−1^, it is a bidentate ligand. When Δ values is from 138 cm^−1^ to 200 cm^−1^, it is considered a bridging ligand [19]. This indicates that the composite material is a bridging ligand material. The peaks at 1625 cm^−1^ and 1506 cm^−1^ correspond to C-C vibrations in H_2_BDC’s aromatic rings.

Figure 2c shows the thermogravimetric analysis (TGA) of the MIL-101 and MIL-101/GO-n samples. Both materials exhibit a three-stage decomposition pattern, as documented in prior research [20]. The first mass reduction occurs around 373 K, corresponding to the release of adsorbed moisture. A minor weight decrease observed between 473 and 573 K likely stems from residual solvent molecules breaking down within the porous structure. The most substantial thermal degradation takes place from 673 to 773 K, marking the point where the MIL-101 framework itself begins to decompose [20,21].

Significant thermal stability is critical for practical industrial applications and adsorbent regeneration [22]. In typical temperature-swing adsorption (TSA) processes for recovering volatile organic compounds (VOCs) such as C_3_F_8_, regeneration temperatures are typically maintained below 473 K [15,23]. The MIL-101 framework remains intact at approximately 673 K, providing sufficient thermal safety margin for multiple regeneration cycles and ensuring structural integrity during long-term operation. Furthermore, the TGA curve of the MIL-101/GO-n composite reveals a decomposition onset temperature nearly identical to that of pristine MIL-101 (Figure 2c). This indicates that the introduction of GO does not adversely affect the inherent thermal stability of the MIL-101 framework. Such high thermal stability is a critical prerequisite for applying these adsorbents in real industrial environments, where high stability ensures their long-term durability and reusability.

**Figure 3:**
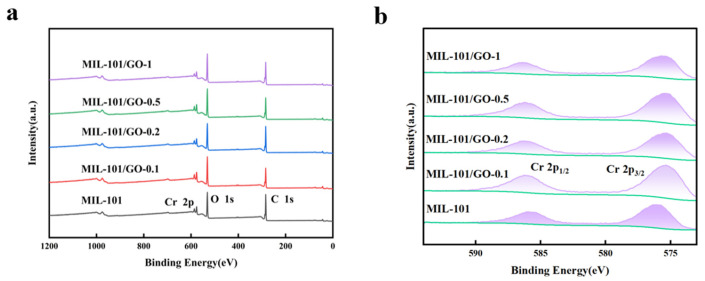
(**a**) Full XPS spectra of MIL-101 and MIL-101/GO-n, (**b**) Cr 2p.

The X-ray photoelectron spectrometers of the five samples are shown in Figure 3. Figure 3a demonstrates that MIL-101 is primarily composed of carbon, oxygen, and chromium. The Cr 2p (Figure 3b) reveals two prominent peaks at 577.2 eV and 587.3 eV, which are characteristic of Cr 2p_3/2_ and 2p_1/2_ orbitals. These indicate the presence of trivalent chromium within the MIL-101 framework, consistent with previous studies [24,25,26]. In addition, the peaks of Cr in the composites are shifted compared to MIL-101, which suggests that oxygen-containing functional groups in GO form coordination bonds with trivalent chromium, leading to a change in electron density around the Cr centers [26].

**Figure 4:**
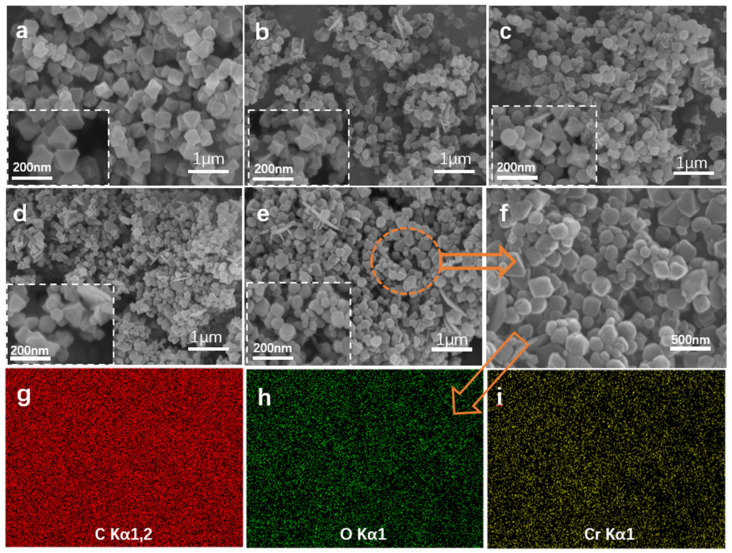
Electron microscope images of MIL-101 and MIL-101/GO-n: (**a**) MIL-101, (**b**) MIL-101/GO-0.1, (**c**) MIL-101/GO-0.2, (**d**) MIL-101/GO-0.5, (**e**) MIL-101/GO-1 (1 μm), (**f**) MIL-101/GO-1 (500 nm) MIL-101/GO-1 elemental energy spectrum diagrams: (**g**) C spectrum, (**h**) O spectrum, (**i**) Cr spectrum.

Figure 4a–f shows Electron microscope images of the MIL-101 and composites. Figure 4b shows that MIL-101/GO-0.1 exhibits an octahedral shape that mirrors that of MIL-101 (Figure 4a). As GO concentration rises, the composite particles shrink in size and develop increasingly uneven shapes, which is visible in Figure 4c–e. For MIL-101/GO-1, these features are more pronounced (Figure 4e). As mentioned previously [16], this phenomenon likely stems from the interaction between oxygen-based functional groups in graphene oxide (GO) and the chromium (III) sites within MIL-101, effectively suppressing the crystal growth of the composites. The uniform dispersion of carbon, oxygen, and chromium elements across the MIL-101/GO-1 composite, as evidenced by EDS mapping in Figure 4g–i, further supports this explanation.

**Figure 5:**
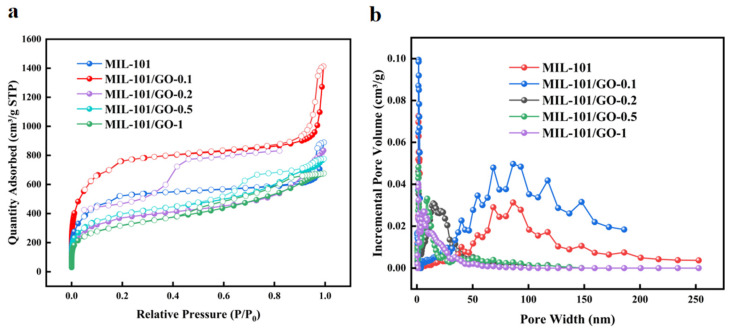
(**a**) N_2_ adsorption–desorption isotherms of composites at 77 K. (Solid symbols indicate adsorption isotherms and hollow symbols indicate desorption isotherms.), (**b**) pore size distribution of MIL-101 and MIL-101/GO-n.

Figure 5a shows the N_2_ adsorption–desorption isotherms of MIL-101 and MIL-101/GO-n samples at 77 K, revealing characteristic Type I behavior with hysteresis loops that confirm bimodal microporosity [14,27]. Pore size distributions of MIL-101 and MIL-101/GO-n (Figure 5b) indicate that composites and MIL-101 maintain a microporous structure, while quantitative analysis (Table 1) shows that MIL-101/GO-0.1 exhibits enhanced porosity than that of MIL-101—notably exhibiting maximal Langmuir (4021 m^2^/g) and BET (2755 m^2^/g) surface areas with a total pore volume of 2.18 cm^3^/g. Conversely, higher GO doping (MIL-101/GO-0.2, -0.5, -1) shows progressive textural degradation, attributed to competing mechanisms: interfacial pore generation at MIL-101/GO boundaries compensates for GO’s non-porous nature at low concentrations [28,29], whereas excessive GO (>0.1 wt%) induces structural distortion that disrupts framework integrity, blocks pore channels, and reduces accessible surface area. Consequently, doping 0.1 wt% GO loading maximizes porosity enhancement, while higher concentrations exert net detrimental effects on gas adsorption functionality. This implies that a further increase in GO content would not have a favorable effect.

### 3.2. Adsorption Isotherms of C_3_F_8_ on MIL-101 and MIL-101/GO-n

**Figure 6:**
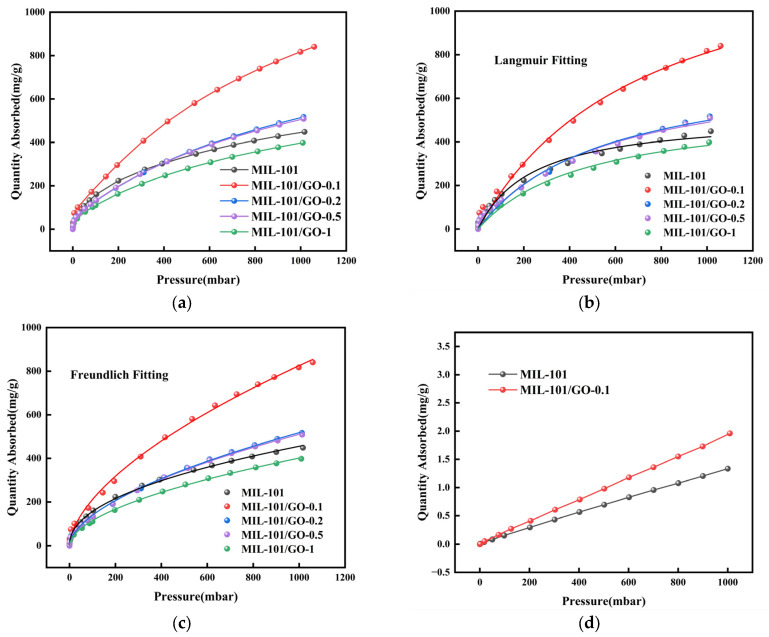
(**a**) Isotherms of C_3_F_8_ adsorption on MIL-101 and MIL-101/GO-n, (**b**) C_3_F_8_ adsorption Langmuir model fitting on MIL-101 and MIL-101/GO-n, (**c**) C_3_F_8_ adsorption Freundlich model fitting on MIL-101 and MIL-101/GO-n, (**d**) isotherms of N_2_ adsorption on MIL-101 and MIL-101/GO-0.1.

Figure 6a shows the isotherms of C_3_F_8_ and N_2_ on MIL-101 and MIL-101/GO-n samples. In the low-pressure region, the uptake of C_3_F_8_ increases dramatically with increasing pressure—a phenomenon driven by microporous adsorption mechanisms. The adsorption capacity of C_3_F_8_ on MIL-101/GO-0.1 can reach up to 840.5 mg/g at 298 K, which is an 87% improvement over the capacity of pure MIL-101. Moreover, this adsorbent’s performance leaves some other well-known adsorbents in the dust, e.g., 13X, CZR-100, β molecular sieves [30], shown in Table 2.

In order to enhance comprehension of the adsorption mechanism, the adsorption isotherms of C_3_F_8_ were fitted by the Langmuir and the Freundlich adsorption models, as shown in Figure 6b,c. The Freundlich model fits the adsorption isotherms of MIL-101 and MIL-101/GO-0.1 very well, and the correlation coefficients R_2_ are up to 0.9989 (for MIL-101) and 0.9984 (for MIL-101/GO-0.1) at 298 K, respectively. The Freundlich model implies that the MIL-101/GO-0.1 possesses a diverse array of heterogeneous active sites, exhibiting multilayer adsorption behavior. This structural complexity, featuring multiple active site types, strengthens the interaction between C_3_F_8_ and MIL-101/GO-0.1, enhancing its adsorption capacity [31]. However, it is important to note the limitations of this empirical model: while it describes the data well, it does not yield fundamental thermodynamic parameters such as the maximum monolayer capacity or the adsorption energy. The Langmuir model, in contrast, showed a slight deviation, particularly at lower pressures, reinforcing the notion of a non-ideal, heterogeneous adsorption surface. The superior fit of the Freundlich model underscores the enhanced heterogeneity and multiplicity of binding sites in the composite, which contributes to its strong, gradient-dependent interaction with C_3_F_8_ molecules.

### 3.3. Breakthrough Experiment

**Figure 7:**
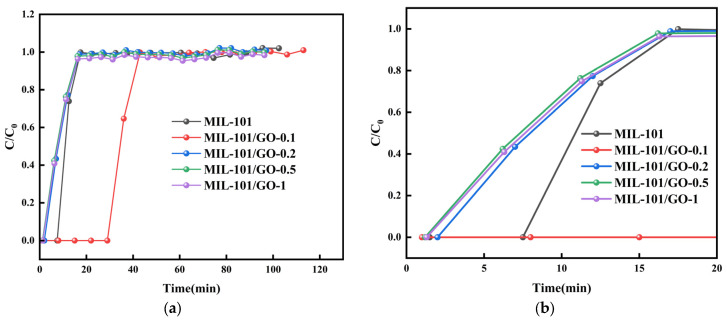
(**a**) Breakthrough curves of C_3_F_8_ on MIL-101 and MIL-101/GO-n, (**b**) Breakthrough curves of C_3_F_8_ on MIL-101 and MIL-101/GO-n (period of breakthrough).

Figure 7 shows the breakthrough curve of C_3_F_8_ on MIL-101 and composites. The MIL-101’s breakthrough time is 7.5 min. The composites exhibit varying adsorption characteristics as the graphene oxide concentration escalates. The composites’ breakthrough times are 22.5 min, 2.5 min, 1.8 min, and 1.5 min, respectively. It is noteworthy that the time of MIL-101/GO-0.1’s breakthrough and saturation are 29.5 and 43.5 min, which are about 3.93 times and 3.48 times those of MIL-101. This suggests that MIL-101/GO-0.1 possesses the best performance in C_3_F_8_/N_2_ separation, because of the doping of graphene oxide greatly increases the specific surface area and enhances the competitive adsorption of N_2_ with MIL-101/GO-0.1. Furthermore, the doping of GO with dense atomic arrays enhances the surface dispersion force of MIL-101/GO-0.1. The maximum dynamic adsorption capacity of C_3_F_8_ by MIL-101/GO-0.1 is 210 mg/g, which is 5.45 times higher than that of the unmodified MIL-101.

The above analysis indicates that the doping of an appropriate amount of graphene oxide can improve the adsorption performance of the MIL-101. However, the presence of excessive GO in the reactants instead leads to a decrease in the adsorption capacity of the composites. The presence of excessive graphene oxide in the reactant impedes the coordination between the metal ions of chromium and H_2_BDC, which leads to a collapse in the morphology of the composites, as evidenced by the result of the SEM images of the composites [16].

### 3.4. Heat of Adsorption of C_3_F_8_ on MIL-101 and MIL-101/GO-0.1

**Figure 8:**
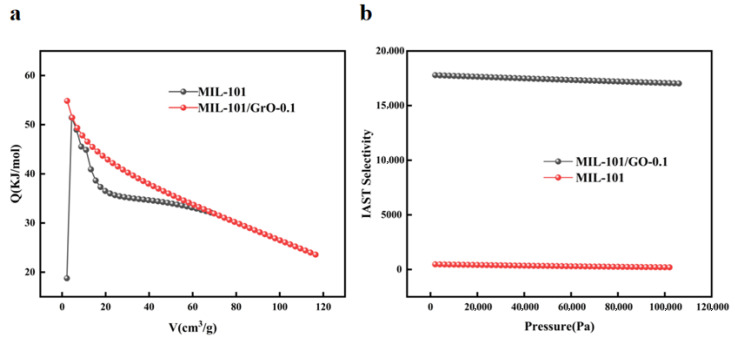
(**a**) Heat of adsorption of C_3_F_8_ by MIL-101 and MIL-101/GO-0.1, (**b**) IAST separation selectivity of C_3_F_8_/N_2_ on MIL-101 and MIL-101/GO-0.1.

Figure 8a shows the heat of adsorption of C3F8 on MIL-101 and MIL-101/GO-0.1, and Figure 8b shows the IAST separation selectivity of C_3_F_8_/N_2_ on these materials. The Clausius–Clapeyron equation was used to figure out the isothermal heat of adsorption, and it turns out that the heat of adsorption of MIL-101 is about 33.37 kJ/mol. And the heat of adsorption of MIL-101/GO-0.1 is higher at the lower adsorption amount of MIL-101/GO-0.1, which is 54.85 kJ/mol, decreasing gradually thereafter. This indicates that the modified MIL-101/GO-0.1 has a higher affinity for C_3_F_8_, which can be attributed to the fact that the modified MIL-101/GO-0.1 has a larger specific surface area and pore volume, which provides more active adsorption sites, and at the same time, the enhance of surface dispersion of MIL-101/GO-0.1 makes it easier to bind MIL-101/GO-0.1 to C_3_F_8_.

According to the ideal adsorption solution theory (IAST), the selectivity for separation in the C_3_F_8_/N_2_ mixture was calculated as shown in Figure 8b. MIL-101/GO-0.1 demonstrates greater adsorption selectivity than MIL-101 alone. And MIL-101/GO-0.1’s IAST separation selectivity for C_3_F_8_/N_2_ reaches 17,069, which is 84.03 times higher than that of MIL-101 (203). The exceptionally high selectivity indicates that during actual adsorption processes, MIL-101/GO-0.1 prioritizes the adsorption of C_3_F_8_ over N_2_.

However, it is worth noting that the IAST model is founded on key idealizations, including the assumptions of an adsorbent surface with homogeneous energy distribution and negligible adsorbate–adsorbate interactions in the adsorbed phase. In practice, surface heterogeneity and molecular interactions may cause deviations between IAST predictions and actual mixture adsorption, typically leading to somewhat lower experimental selectivity in dynamic breakthrough experiments. Therefore, the reported IAST value primarily serves as a robust indicator of intrinsic separation potential under ideal conditions.

### 3.5. Adsorption Cycle Performance Test

**Figure 9:**
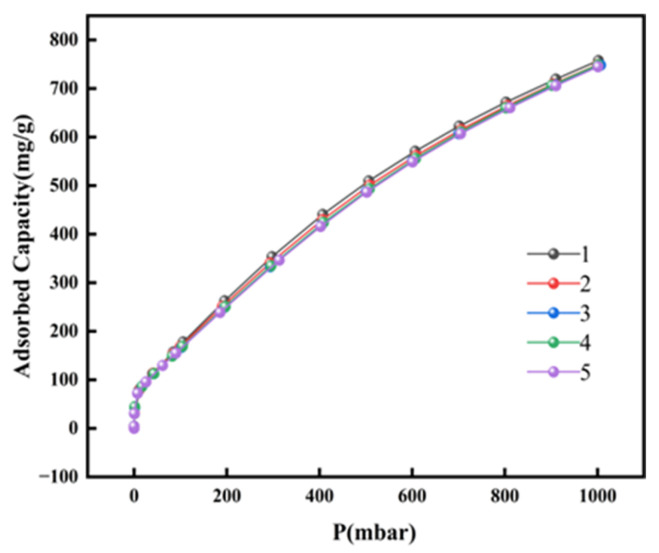
MIL-101/GO-0.1 adsorption isotherms for five consecutive cycles of C_3_F_8_.

Figure 9 shows the adsorption isotherms of MIL-101/GO-0.1 for five cycles of C_3_F_8_. The result of five cycles shows that MIL-101/GO-0.1 has almost the same adsorption capacity for C_3_F_8_. However, the adsorption capacity decreases from the initial 840.5 mg/g to 757.5 mg/g, which is potentially due to the adsorption of the difficult-to-desorb gases in the air. MIL-101/GO-0.1 can still maintain 90% of its initial adsorption capacity after five cycles, which indicates that MIL-101/GO-0.1 has good adsorption stability.

## 4. Discussion

The superior C_3_F_8_ adsorption and separation performance of the MIL-101/GO-0.1 composite stems from a synergistic enhancement of its textural and surface chemical properties. The preservation of the MIL-101 framework provides optimally sized micropores (0.59 nm) for the confinement of C_3_F_8_ molecules (0.55 nm) [32]. Concurrently, doping GO increases surface area, introduces structural heterogeneity, and likely generates additional defect sites. This creates a more heterogeneous adsorption landscape with enhanced dispersive interactions, as evidenced by the excellent fit to the Freundlich model and the dramatic boost in capacity and selectivity [33].

To translate this promising performance into practical application, key challenges must be acknowledged and addressed. First, the extraordinary theoretical (IAST) selectivity requires validation through long-term mixed-gas breakthrough tests with realistic industrial feed compositions, including potential trace contaminants like moisture. Second, while the acid-free synthesis is advantageous, its scalability and the associated cost of achieving uniform GO dispersion at larger scales need evaluation. Finally, future work must focus on engineering the powder into robust, structured forms (e.g., pellets) and conducting comprehensive process engineering studies to assess cycle efficiency, regeneration energy demands, and overall techno-economic viability. Addressing these points is crucial for advancing this material from a laboratory proof-of-concept to a viable industrial adsorbent.

## 5. Conclusions

In summary, a series of MIL-101/GO composites was successfully synthesized via a green, acid-free solvothermal route. The MIL-101/GO-0.1 exhibited significantly enhanced textural properties and surface heterogeneity, leading to exceptional performance for C_3_F_8_ capture: a high adsorption capacity (840.5 mg/g), a remarkable IAST selectivity over N_2_ (17,069), and robust cyclic stability. These improvements are attributed to the synergistic combination of optimal pore confinement and enhanced adsorbent-adsorbate interactions.

Despite these promising results, several challenges must be addressed to advance toward practical application. The study’s limitations include the need to validate the theoretical selectivity under realistic, mixed-gas industrial conditions and to assess long-term stability against potential feed impurities. Future work should focus on scaling the synthesis, formulating the powder into engineered structures, and conducting a comprehensive techno-economic analysis.

This work establishes GO doping as an effective strategy for tailoring MOF properties for challenging gas separations and positions MIL-101/GO-0.1 as a promising candidate for the recovery of high-GWP fluorocarbons.

## Figures and Tables

**Table 1:** Pore structure parameters of the MIL-101 and MIL-101/GO-n.

Samples	Surface Area (m^2^/g)		Pore Volume (cm^3^/g)		
	Langmuir	BET	Micropore	Mesopore	Total Pore
MIL-101	2878	1905	0.79	0.59	1.38
MIL-101/GO-0.1	4021	2755	1.14	1.04	2.18
MIL-101/GO-0.2	2879	1341	0.44	0.85	1.29
MIL-101/GO-0.5	2949	1438	0.37	0.83	1.20
MIL-101/GO-1	2658	1151	0.22	0.92	1.14

**Table 2:** Comparison of C_3_F_8_ adsorption results with common materials at 298 K.

Adsorbent	Temperature (K)	Pressure (Pa)	C_3_F_8_ Capacity (mg/g)	Reference
MIL-101/GO-0.1	298	180	52.426	This work
MIL-101(Cr)	298	180	26.534	This work
13X	298	180	6.335	[28]
β-Molecular Sieve	298	180	3.948	[28]
CZR-100	298	180	7.144	[28]

## Data Availability

The original contributions presented in this study are included in the article. Further inquiries can be directed to the corresponding authors.

## Abbreviation

The following abbreviation is used in this manuscript:

IAST	Ideal adsorption solution theory
